# Protein-protein docking on hardware accelerators: comparison of GPU and MIC architectures

**DOI:** 10.1186/1752-0509-9-S1-S6

**Published:** 2015-01-21

**Authors:** Takehiro Shimoda, Shuji Suzuki, Masahito Ohue, Takashi Ishida, Yutaka Akiyama

**Affiliations:** 1 Department of Computer Science, Graduate of Information Science and Engineering, Tokyo Institute of Technology, 2-12-1 W8-76, Ookayama, Meguro-ku, 152-8550 Tokyo, Japan; 2 Japan Society for the Promotion of Science (JSPS) Research Fellow, Japan; 3 Education Academy of Computational Life Sciences (ACLS), Tokyo Institute of Technology, 2-12-1 W8-93, Ookayama, Meguro-ku, 152-8550 Tokyo, Japan

**Keywords:** GPU, MIC, Intel Xeon Phi, fast Fourier transform, protein-protein docking, structural biology

## Abstract

**Background:**

The hardware accelerators will provide solutions to computationally complex problems in bioinformatics fields. However, the effect of acceleration depends on the nature of the application, thus selection of an appropriate accelerator requires some consideration.

**Results:**

In the present study, we compared the effects of acceleration using graphics processing unit (GPU) and many integrated core (MIC) on the speed of fast Fourier transform (FFT)-based protein-protein docking calculation. The GPU implementation performed the protein-protein docking calculations approximately five times faster than the MIC offload mode implementation. The MIC native mode implementation has the advantage in the implementation costs. However, the performance was worse with larger protein pairs because of memory limitations.

**Conclusion:**

The results suggest that GPU is more suitable than MIC for accelerating FFT-based protein-protein docking applications.

## Introduction

Many recently developed hardware accelerators, such as ClearSpeed, Cell Accelerator Board, and GRAPE, were developed for specific purposes, but graphics processing units (GPUs) have currently become the most popular because of their excellent performance and simple programming environments, such as NVIDIA's CUDA and OpenCL [1].

Many integrated core (MIC) architectures are hardware accelerators developed by Intel, which have been released recently as the Xeon Phi co-processor. Similar to a GPU, MIC includes many tiny computing cores. However, the core can be used in the same ways as a general CPU core. MIC is one of the main architectures used in current supercomputing systems [2]. For example, Tianhe-2 at the National Super Computer Center in Guangzhou, China, has 48,000 MIC boards and it was the "fastest" supercomputer in the world in June 2014 [3]. However, the TOP500 ranking only shows that MIC has good performance when solving LINPACK benchmark problems and it is still not known whether MIC can accelerate real applications, because acceleration depends on the nature of the application and the accelerator.

Thus, the actual applications should be considered during evaluations of hardware accelerators.

At present, various applications have been mapped onto accelerators. In particular, GPU-based applications have been developed in various research fields, including genome analysis [4,5], molecular dynamics simulations [6,7], and quantum chemistry calculations [8,9]. By contrast, only a few MIC applications have appeared, such as molecular dynamics simulations [10] and genome-wide association studies [11], which means that it is difficult to compare the performance of GPU and MIC in real applications based on previous studies.

In the present study, we evaluated the performance of GPU and MIC using protein-protein docking calculations, which is a real-world application in computational biology. Protein-protein docking is a method used to predict protein complex structures based on monomeric protein structures. At present, the most popular docking methods employ rigid-body docking using a voxel-based representation in a three-dimensional (3D) grid space with a discrete convolution-based scoring function, where the fast Fourier transform (FFT) is employed to speed up the calculations [12-14]. FFT-based protein-protein docking needs only a few minutes to compute a protein pair, although the performance is not adequate for large-scale interactome predictions, which require docking calculations for millions of protein pairs. Thus, further acceleration is still required.

In this study, we used MEGADOCK [15,16], which is a FFT-based protein- protein docking program developed by our group, and we mapped the docking calculations onto GPU and MIC. Next, we compared the acceleration obtained with these accelerators and evaluated the best method for the acceleration of FFT-based real-world applications. In addition to the computational performance, we also considered the implementation costs.

## MEGADOCK

MEGADOCK is a protein-protein interaction prediction system that uses FFT-based protein-protein docking based on the Katchalski-Katzir algorithm [12]. MEGADOCK was implemented in C++. MEGADOCK evaluates each docking pose based on three types of score function, i.e., shape complementarity, electrostatic interactions, and the desolvation free energy. It calculates these functions using a single FFT calculation and is much faster than the well-known docking program ZDOCK [13], which requires eight FFT calculations. MEGADOCK has already been parallelized using MPI and OpenMP for multiple combinations of protein pairs [16,17].

**Figure 1:**
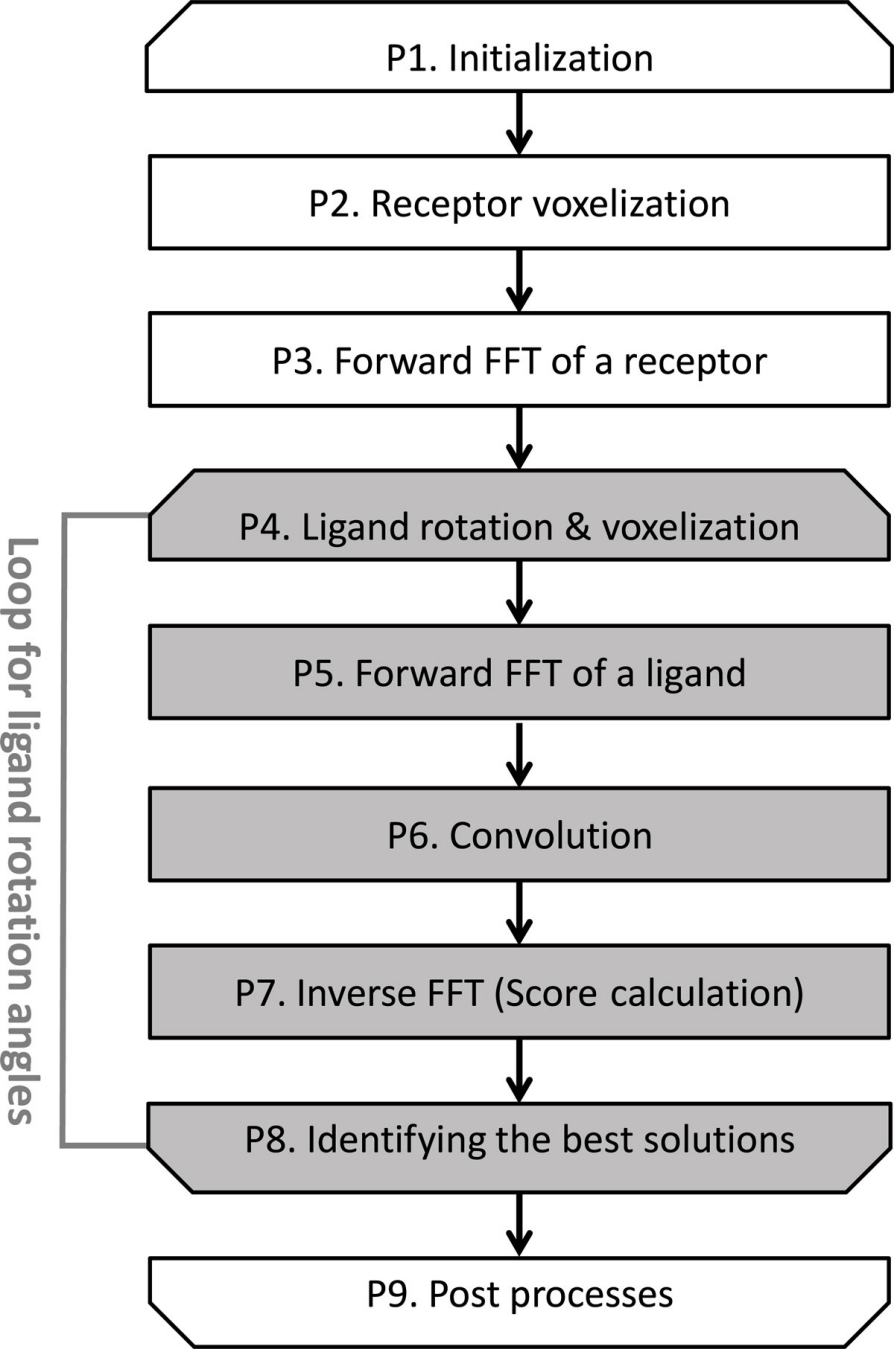
**Process flow of FFT-based protein-protein docking tools**.

Figure 1 shows the flow of the docking processes in MEGADOCK, where the flow is based mainly on the Katchalski-Katzir algorithm. In the Katchalski-Katzir algorithm, the pseudo-interaction energy score (the docking score *S*) between a receptor protein and a ligand protein is calculated as the convolution of two discrete functions using *N*^3^-point forward FFT and inverse FFT (IFFT), as follows:

(1) $$S\left(t\right)=\underset{v\in V}{\sum }R\left(v\right)L\left(v+t\right)$$

(2) =IFFT[FFT[R(v)]*FFT[L(v)]],

where *R *and *L *are the discrete score functions of the receptor and ligand proteins, respectively, ***v ***is a coordinate in the 3D grid space  V, ***t ***is the parallel translation vector of the ligand protein, *∗ *is the complex conjugation operator, and *N *, which is referred to as the FFT size, is double the size of the grid. The discrete score functions *R *and *L *are based on shape complementarity (rPSC model [15]), electrostatic interactions (FTDock potential [18]) and desolvation free energy (RDE model [19]). To identify the best docking pose, the possible ligand orientations are examined exhaustively at *n_θ _*rotation angles with a given step size *θ*. For each rotation, the ligand protein is translated into *N*/2 × *N*/2 × *N*/2 voxels in the  V grid space (where *N*/2 is an edge of  V). The decoy (relative conformation model of the receptor and ligand) that yields the highest value of *S *for each rotation is recorded. In this method, a total of *n*_*θ *_× *N*^3 ^docking poses are evaluated for one protein pair. To execute the simple convolution sums in eq. (1) directly, $O\left(N^{6}\right)$ calculations are required, although this is reduced to $O\left(N^{3}\text{log}N\right)$ using the FFT in eq. (2). The FFT-based docking calculation comprises the following processes: initialization (P1), receptor voxelization (P2), forward FFT of a receptor (P3), ligand voxelization (P4), forward FFT of a ligand (P5), convolution (P6), inverse FFT (P7), identifying the best solutions (P8), and post processes (P9), as shown in Figure 1. Processes (P4)-(P8) are looped *n_θ _*times. MEGADOCK uses an *n_θ _*value of 3,600 as the default setting. Table 1 shows the proportions of the docking calculation time required by each process in MEGADOCK. This profile was obtained based on the docking calculation of a protein complex (Protein Data Bank (PDB) [20] ID: [PDB:1JK9]; receptor: CCS metallochaperone (243 residues), ligand: SOD1 superoxide dismutase (153 residues)). The FFT size *N *of the docking calculation was 128, which is typical in the current protein structure database. The profile was obtained using an Intel Xeon E5-2670 2.60 GHz, one CPU core. FFT processes (P5 and P7) accounted for most of the processing time (75.9%). However, other calculations, such as voxelization and identifying the best solutions, still accounted for considerable proportions of the total time.

**Table 1:** Docking calculation time profile using a one CPU core (PDB ID: [PDB:1JK9]).

	Time [sec.]	Ratio [%]
P1. Initialization	0.0	0.0
P2. Receptor voxelization	0.3	0.2
P3. Forward FFT of receptor	0.1	0.0
P4. Ligand rotation & voxelization	12.9	6.9
P5. Forward FFT of ligand	69.8	37.5
P6. Convolution	27.4	14.7
P7. Inverse FFT	71.5	38.4
P8. Identifying best solutions	4.3	2.3
P9. Post processes	0.0	0.0

Total	186.4	100.0

## GPU implementation

To compare GPU and MIC, the MEGADOCK program should be mapped onto both GPU and MIC. The MIC implementation was newly implemented for this study. For GPU implementation, we used MEGADOCK-GPU developed in the previous study [21]. In this section, we provide a brief description of the GPU implementation. We implemented the following processes on a GPU: forward FFT of a receptor (P3), ligand rotation and voxelization (P4), forward FFT of a ligand (P5), convolution (P6), inverse FFT (P7), and identifying the best solutions (P8). The details of each implementation are described in the following sections.

In ligand rotation process (P4), the atomic coordinates of a ligand are updated according to a given rotation matrix. The process is independent for each atom and it can be fully parallelized. We mapped the atomic coordinates onto a GPU. In ligand voxelization process (P4), MEGADOCK sets a suitable rPSC score, electrostatic interaction values, and desolvation free energy scores for the ligand voxel model during this process. Ligand voxelization calculates the distance between the coordinates of an atom and each grid, before assigning a value to each grid within the van der Waals radius of the atom. The assignment process can be parallelized for each atom. The rPSC score and the desolvation free energy score of a ligand has only binary states (0 or 1), and the electrostatic interaction value of a grid is calculated as the cumulative sum of the values of all adjacent atoms, thus the calculation order for each atom can be exchanged freely. Therefore, we processed the atoms in parallel and mapped them onto a GPU. Thus, multiple atoms were processed simultaneously on different GPU cores in this process. In FFT processes (P3, P5, P7), single precision complex 3-dimentional FFT is performed using the NVIDIA cuFFT library to map the FFT calculations onto a GPU. In convolution process (P6), the output of FFT of receptor voxel is complex conjugated and multiplied by the output of FFT of ligand voxel. The convolution can be independent for each grid, thus we mapped them onto a GPU. In identifying the best solutions process (P8), the best docking pose was selected according to the docking score. This process was also implemented on a GPU using reduction.

In our implementation, the transfer of large volumes of data from a host to a GPU occurred only once. These data comprised the original atom coordinates of a ligand and the Fourier transformed receptor grid information, which were transferred first. Only trivial volumes of data transfer were required (12 bytes for angular information and 8 bytes for the calculation results) in the loop for each ligand rotation angle.

### Rotation of the ligand

In this process, the atomic coordinates of a ligand are updated according to a given rotation matrix. The process is independent for each atom and it can be fully parallelized. We mapped the atomic coordinates onto a GPU.

### Ligand voxelization

MEGADOCK sets a suitable rPSC score, electrostatic interaction values, and desolvation free energy scores for the ligand voxel model during this process. Ligand voxelization calculates the distance between the coordinates of an atom and each grid, before assigning a value to each grid within the van der Waals radius of the atom. The assignment process can be parallelized for each atom. The rPSC score and the desolvation free energy score of a ligand has only binary states (0 or 1), and the electrostatic interaction value of a grid is calculated as the cumulative sum of the values of all adjacent atoms, thus the calculation order for each atom can be exchanged freely. Therefore, we processed the atoms in parallel and mapped them onto a GPU. Thus, multiple atoms were processed simultaneously on different GPU cores in this process.

### Forward and inverse FFT

We used the NVIDIA cuFFT library [22] to map the FFT calculations onto a GPU.

### Convolution

The convolution can be independent for each grid, thus we mapped them onto a GPU.

### Identifying the best solutions

In this process, the best docking pose was selected according to the docking score. This process was also implemented on a GPU using reduction.

### Data transfer

In our implementation, the transfer of large volumes of data from a host to a GPU occurred only once. These data comprised the original atom coordinates of a ligand and the Fourier transformed receptor grid information, which were transferred first. Only trivial volumes of data transfer were required (12 bytes for angular information and 8 bytes for the calculation results) in the loop for each ligand rotation angle.

## MIC implementation

The MIC architecture can be used in two different modes. In the offload mode, only specific sections of the program are executed on the MIC and the user has to add pragmas in the code to organize the data transfer and parallelization. In the native mode, the program is executed on the MIC alone and there is no need to change the source code of existing applications if the program is parallelized using OpenMP.

We accelerated protein-protein docking calculations by utilizing MIC in both the offload and native modes. MEGADOCK has already been parallelized by looping the rotational angles of the ligand protein using OpenMP and it can be run in parallel on a multi-core CPU. Thus, the native mode implementation parallelizes the looping of the rotational angles, whereas the offload mode implementation is parallelized during each docking calculation process.

### Offload mode

In the offload mode implementation, we mapped the most intensive processes, i.e., from (P4) to (P8), onto the MIC architecture in a similar manner to the GPU implementation. Processes such as ligand atom rotation, voxelization, and convolution were parallelized in the same manner as the GPU implementation with OpenMP. The process used to identify the best solution was accelerated with the "reduction" pragma in OpenMP. The Intel Math Kernel Library (MKL) was used to perform the FFT calculations. On MIC, the FFT calculations were parallelized automatically and the number of threads was optimized to the FFT size.

### Native mode

**Figure 2:**
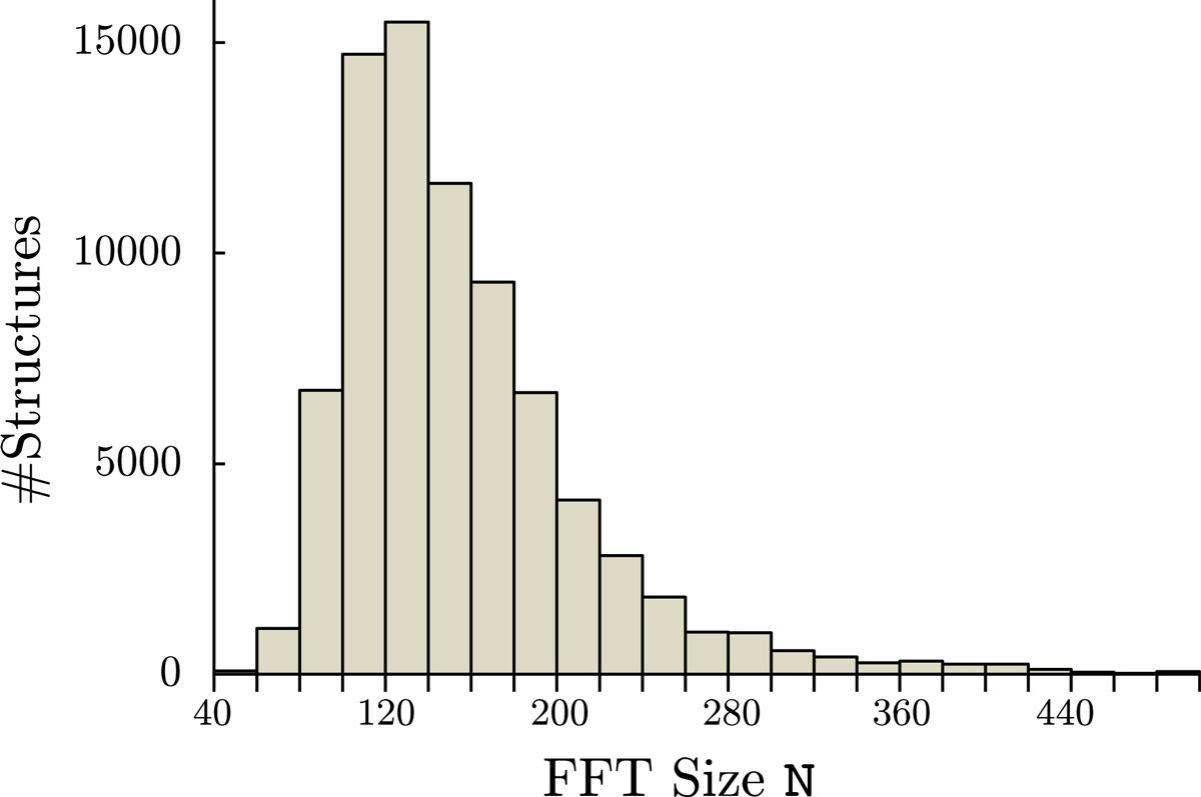
**FFT size of the proteins registered in the PDB (experimentally determined by X-ray diffraction, 78,958 structures)**.

In the native mode implementation, we did not change the program, but added the compile option. In contrast to the offload mode and the GPU implementation, the native mode implementation did not require data transfer from the host to the MIC. However, the serial sections in the program may have operated more slowly compared with that when running on a CPU because the clock of the MIC cores was slower than that of the CPU. In addition, a problem occurred in the native mode when the docking target was a large protein pair. This was because the parallelization of the loop used for rotational angles required per-thread memory. Thus, the largest memory requirements were for the input and output of the FFT, which were specific to each thread. The FFT requirements were:

$$\begin{array}{c} \text{Memory requirements for FFT on one thread}\\ =\left(\text{input array}+\text{output array}\right)\\ \;\;\;\;\times \;\text{size of complex float type}\times N^{\text{3}}\\ =16N^{3}\;\text{bytes} \end{array}$$

on each thread, where *N *is the number of 3D FFT points. Thus, if all 240 threads were used, 240 × 16*N*^3 ^bytes of memory were required. However, the Xeon Phi 5110P, which is a product of MIC architecture, has an onboard memory of 8 GB. Therefore, *N *could be up to 127 because $N=\sqrt[{3}]{8\times 10^{9}\text{ bytes}/240\text{ threads}/16}=127.7\ldots$ (this was actually smaller). Thus, even if we only used 60 threads, the FFT size would have been less than 202, because $N=\sqrt[{3}]{8\times 10^{9}\text{ bytes}/60\text{ threads}/16}=202.7\ldots$. Unfortunately, the FFT sizes of many proteins exceed 127. Figure 2 shows the distribution of the FFT sizes of proteins that are experimentally determined by X-ray diffraction and registered in the PDB (as of April 16, 2013). For over 46.8% of the proteins, the current MIC specification would not utilize all of the computing cores to perform docking calculations in the native mode.

## Experiments

To evaluate the acceleration of protein-protein docking application using accelerators, we measured the MEGADOCK execution performance using CPUs, a GPU, and a MIC. We also compared the performance of the MIC offload and native modes. We used the following five conditions in the comparisons: docking calculation using one CPU core ("1CPU"), docking calculation using an OpenMP implementation and the eight CPU cores included in a CPU socket ("8CPUs"), GPU-accelerated docking calculation using one GPU and one CPU core ("GPU"), docking calculation accelerated by the MIC offload mode implementation using one MIC and one CPU core ("MIC_offload_"), and docking calculation executed on a MIC using an OpenMP implementation and the MIC native mode ("MIC_native_"). Table 2 shows the difference of parallelization among each implementation.

**Table 2:** Difference of parallelization among GPU and MIC offload and native implementations (*n_th _*is number of MIC threads).

Target of parallelization		#threads used for one ligand angle	Consumption of accelerator memory
GPU	Each process in one ligand angle	All GPU threads	For only one ligand
MIC_offload _	Each process in one ligand angle	All MIC threads	For only one ligand
MIC_native _	Loop of rotational angles of ligand	One MIC thread	For *n_th _*ligands

To evaluate the time required for the calculations in each condition, we performed docking calculations for the same dataset using each system. We used the gettimeofday() function to measure the calculation time.

All of the conditions were compared based on three metrics: the performance with large-scale benchmark dataset, the performance with protein pairs of different sizes, and the acceleration rate of each process. For large proteins, as mentioned earlier, it was impossible to execute docking calculations using the MIC native mode implementation with all of the MIC cores because of the memory limitations of the Xeon Phi coprocessor. Thus, the number of threads was adjusted according to the protein size when we used the MIC native mode.

### Computational environment

The specifications of the computation nodes are shown in Table 3. The CPU/MIC node was used to measure the performance of "1CPU," "8CPUs," "MIC_offload_," and "MIC_native_," and the performance of "GPU" was measured using the GPU node.

**Table 3:** Computational environment.

	CPU/MIC node	GPU node
CPU	Intel Xeon E5-2670, 2.60 GHz (8 cores)	Intel Xeon X5670, 2.93 GHz (6 cores)
Memory	54 GB	64 GB
Accelerator	Intel Xeon Phi 5110P, 1.05 GHz (60 cores)	NVIDIA Tesla K20X, 0.73 GHz (2,688 CUDA cores)
Accelerator memory	8 GB	6 GB
OS	CentOS 6.3	SUSE LES 11 SP1
Compiler	Intel C++ Compiler 13.0	Intel C++ Compiler 13.0
FFT library	Intel MKL 11.0	cuFFT (CUDA 5.0)

### Performance evaluation using benchmark dataset

We retrieved 352 protein complex structures from a standard protein-protein docking benchmark set (ZLAB Benchmark 4.0) [23], which contained bound and unbound forms of the protein structures. The proteins sizes were distributed widely in the dataset (from 128 residues to 2,604 residues) and it represented a fairly sampled subset of the current known protein structure complexes.

**Figure 3:**
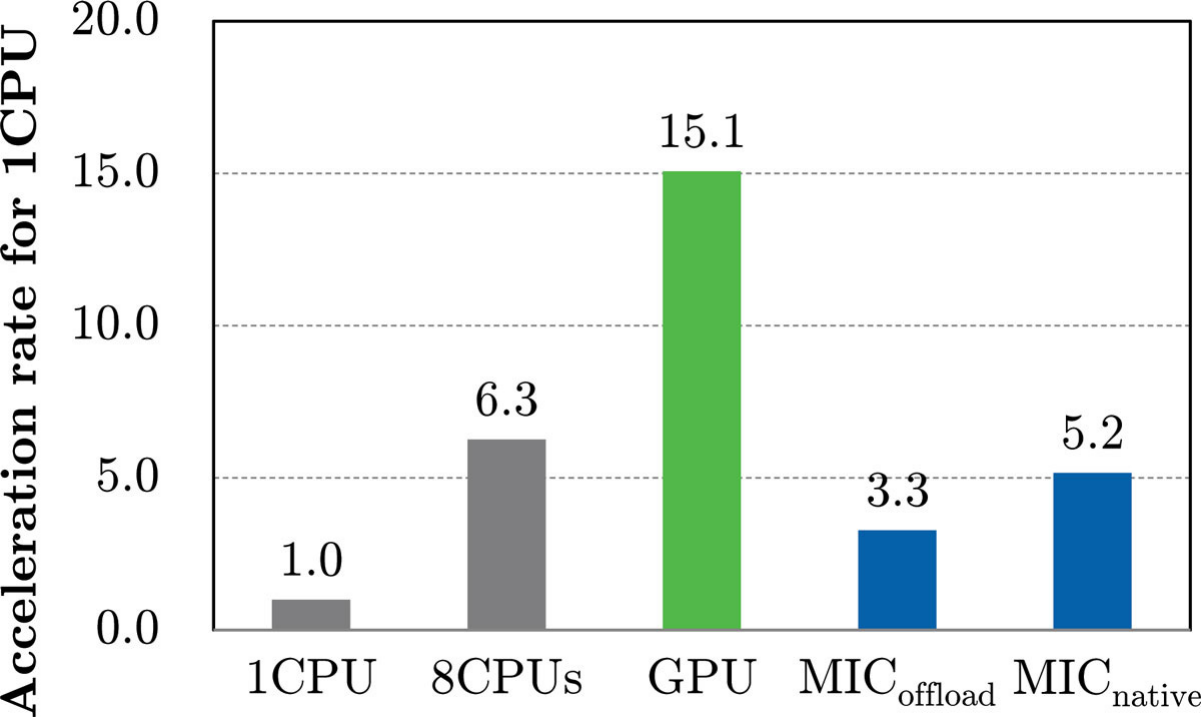
**Acceleration rate for each system based on the total docking calculation time for 352 protein complexes**.

Figure 3 andTable 4 show the total docking calculation time results for the dataset. MEGADOCK was parallelized previously using OpenMP and it provided good acceleration with multicores, as reported in our previous study [17]. With this dataset, it achieved a 6.3-fold speed up using eight CPU cores. GPU and MIC also accelerated the protein docking calculations. Using a GPU, the docking calculations were 15.1 times faster than the calculations with a CPU core alone. With a GPU, the acceleration was more than double that obtained with eight CPU cores, i.e., a CPU socket. By contrast, the acceleration rates were increased by 3.3-fold and 5.2-fold with the MIC offload mode and MIC native mode, respectively, which were much lower than the improvements obtained with the GPU. The MIC native mode was faster than the MIC offload mode but slower than a CPU socket.

**Table 4:** Total docking calculation times for 352 protein complexes.

	1CPU	8CPUs	GPU	MIC_offload_	MIC_native_
Total docking time [hour]	30.8	4.9	2.0	9.4	6.0

### Performance with proteins of different sizes

**Figure 4:**
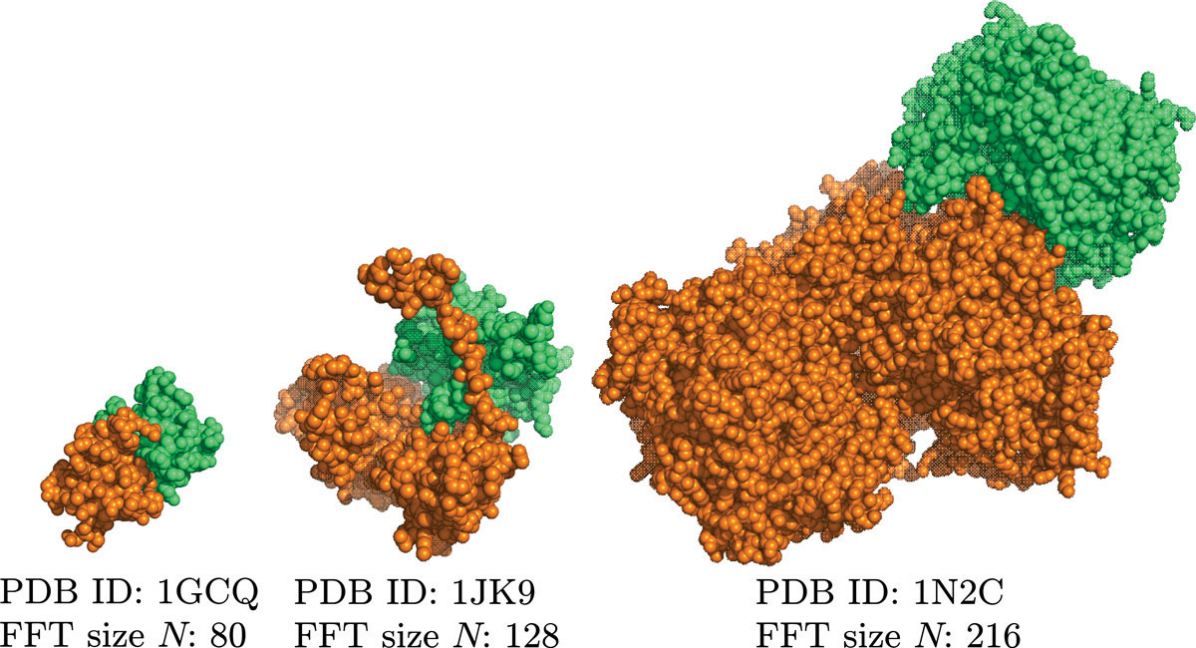
**Images showing protein pairs of different sizes**.

To test the relationship between the FFT size and the speed up with the accelerators, we evaluated the performance with three protein pairs of different sizes. Figure 4 shows images of the three protein pairs, where the orange proteins are receptors and the green proteins are ligands. Table 5 shows the details of each protein and the docking calculation time required for the three protein pairs. The FFT size depended on the size of the protein and "medium" indicates a typical protein and FFT size. The FFT size affected the computational costs directly.

**Table 5:** Docking calculation times and acceleration rates for three proteins of different sizes.

		Small		Medium		Large	
Receptor (#residues)		GRB2 C-ter SH3 domain (61)		CCS metallochaperone (249)		Nitrogenase Mo-Fe protein (2026)	

Ligand (#residues)		Vav N-ter SH3 domain (70)		SOD1 superoxide dismutase (153)		Nitrogenase Fe protein (578)	

PDB ID		[PDB:1GCQ]		[PDB:1JK9]		[PDB:1N2C]	

FFT size		80 × 80 × 80		128 × 128 × 128		216 × 216 × 216	

Docking time [second] (vs. 1CPU)	1CPU	38.3	(1.0×)	186.4	(1.0×)	1105.6	(1.0×)
	8CPUs	8.4	(4.6×)	38.5	(4.8×)	177.5	(6.2×)
	GPU	5.8	(6.6×)	10.8	(17.3×)	62.2	(17.8×)
	MIC_offload_	58.7	(0.7×)	77.0	(2.4×)	180.6	(6.1×)
	MIC_native _	7.6	(5.0×)	26.8	(7.0×)	310.5	(3.6×)

The MIC native mode (MIC_native_) used an optimized numbers of threads, which were the largest numbers available for each protein size (small = 240 threads, medium = 171 threads, and large = 38 threads).

For a small-sized protein pair, the acceleration rates obtained with GPU and MIC were less than those with larger proteins. "GPU" achieved only a 6.6-fold speed up compared with "1CPU" in this case. "MIC_native_" was 5.0 times faster than "1CPU" and it was faster than "8CPUs," but "MIC_offload_" was even slower than "1CPU." This was because the memory offload overhead became large in this case and small FFT calculations were inefficient in the MIC.

For a medium-sized protein pair, the GPU calculation achieved a 17.3-fold speed up compared with the one CPU core, which was much greater than that for a small-sized protein. Because the processes such as FFT and convolution, which show better speedup using accelerators as shown in Table 6 account for larger part of the calculation time in larger protein pairs. The acceleration obtained with MIC was also greater than that with a small-sized protein but it was smaller than "GPU". "MIC_native_" and "MIC_offload_" yielded 7.0-fold and 2.4-fold speed ups compared with "1CPU," respectively. One of the reasons why "MIC_native_" was not effective in accelerating the calculations was that only 171 threads were used because of the memory limitations on the MIC.

**Table 6:** Docking calculation time results for the protein complex (PDB ID: [PDB:1JK9]) for each process (in seconds).

	1CPU	8CPUs		GPU		MIC_offload_		MIC_native_	
P1. Initialization	0.0	0.0		0.8		4.0		0.7	
P2. Receptor voxel	0.3	0.3	(1.1×)	0.3	(1.1×)	0.3	(1.1×)	4.4	(0.1×)
P3. Receptor FFT	0.1	0.1	(1.0×)	0.0	(1.7×)	1.0	(0.1×)	0.3	(0.2×)
P4. Ligand rot & voxel	12.9	3.4	(3.8×)	2.3	(5.5×)	7.4	(1.7×)	1.2	(11.1×)
P5. Ligand FFT	69.8	14.2	(4.9×)	2.2	(31.1×)	15.1	(4.6×)	7.9	(8.9×)
P6. Convolution	27.4	4.6	(5.9×)	1.1	(25.6×)	13.9	(2.0×)	3.6	(7.7×)
P7. Inverse FFT	71.5	14.1	(5.1×)	2.2	(31.8×)	15.2	(4.7×)	8.3	(8.6×)
P8. Identifying the bests	4.3	1.7	(2.5×)	1.7	(2.5×)	9.8	(0.4×)	0.3	(12.5×)
P9. Post processes	0.0	0.0		0.0		0.0		0.0	
Data transfer				0.6		10.1			

Total	186.4	38.5	(4.8×)	10.8	(17.3×)	77.0	(2.4×)	26.8	(7.0×)

The values shown in parentheses are the acceleration rates relative to one CPU core. MIC native used 171 threads.

For large-sized protein pairs, the GPU calculations achieved 17.8-fold speed up compared with the one CPU core and the acceleration rate was almost same as that with a medium-sized protein pair. For a large-sized protein pair, "MIC_native_" was only 3.6 times faster than "1CPU" and it was much smaller compared with the small-sized protein pairs. This was because only 38 threads could be used for the large-sized protein pairs because of the MIC memory limitations. On the other hand, "MIC_offload_" was relatively fast and it achieved a 6.1-fold speed up compared with "1CPU," which was comparable to eight CPU cores.

Overall, GPU implementation delivered the most efficient performance with various protein pair sizes. MIC native mode implementation delivered better performance with small- and medium-sized protein pairs, but its performance was worse with large-sized proteins due to the MIC memory limitations.

### Acceleration rate for each process

Table 6 shows the profile of docking calculation time obtained with each system. The FFT calculations (P5 and P7) were speeded up greatly by the accelerators. In particular, the speed of the FFT calculations on the GPU was over 30 times faster than those on one CPU core. However, the FFT calculations were accelerated much less using the MIC than the GPU, even when using the native mode. This is one of the reasons why the GPU delivered much better acceleration than the MIC. In addition, current MIC systems require more time for data transfer between the host and the accelerator compared with a GPU. Each memory offload by the MIC incurred large overheads and the docking calculations required 3,600 data transfers, although the data size was approximately 200 bytes and there was a small difference in the data transfer speed with the GPU and the MIC. In addition, both the GPU and MIC required the initialization of the accelerators before they could be used, which may have been a bottleneck, especially with small-sized protein pairs.

## Discussion

### Effects of the different computational environments

In this study, we used different nodes to compare the performance of the GPU and MIC. To test MIC, we used a Xeon E5-2670, which was faster than Xeon X5670 used for testing the GPU. However, "GPU" performed almost all of the processes on the GPU so the difference in the performance of the CPU was largely irrelevant with respect to the calculation time. Indeed, the calculation time with "GPU" was almost the same even when we used six CPU cores and one GPU card (9.77 seconds for [PDB:1JK9]). Furthermore, "GPU" achieved the best performance in all of the experiments. Thus, even if X5670 had been replaced with E5-2670, the results of the comparison would not have changed.

### Implementation costs

In addition to the computational speed, the costs of mapping a program onto the accelerators are also important when evaluating the accelerators. Learning a new language to implement a program on accelerators is a demanding task. GPU computing requires a novel programming technique, such as CUDA, whereas a program is ready-to-use with MIC if it is implemented in C, C++, and OpenMP. Thus, we considered the time and effort required to port a code onto a GPU and a MIC, including the offload and native modes.

To map a docking calculation onto a GPU, we had to write several CUDA kernel functions, which describe the processes executed on the GPU, as well as adding statements to facilitate data transfer between the host and the accelerator. We had to add the code with approximately 1,000 lines to the MEGADOCK original code with approximately 7,000 lines. Therefore, the implementation costs were high, although we were familiar with CUDA programming. Furthermore, the source code management costs were increased because the code required many branches and additional source code files for GPU computing.

For the MIC offload mode implementation, we also had to add several pragmas to the offload section so we could execute them on a MIC. This was similar to the GPU implementation but the size of the additional statements was approximately 500 lines, which was less than that required for the GPU implementation.

By contrast, to implement a MIC in the native mode code, we did not need to write any additional code because MEGADOCK had already been parallelized for looping ligand protein rotational angles using OpenMP. We only added a compile option for constructing the MIC native mode binary. Thus, the implementation cost was lowest for the MIC native mode. To execute the program, however, we needed to copy the binary and libraries files, and execute them remotely on each Xeon Phi. Therefore, the operability was more complex than that with the other systems.

## Conclusion

In this study, we compared the acceleration obtained by applying GPU and MIC to protein-protein docking calculation, which is a FFT-based real-world application. GPU computing required considerable effort to map the calculations but it achieved the best performance. The MIC offload mode implementation had similar costs to GPU but its performance was far inferior to that obtained with the GPU. With the GPU implementation, the protein-protein docking calculations were completed about five times faster than the MIC offload mode implementation. The MIC native mode implementation had the advantage that the user did not have to write additional code, but this was mainly because the program code had already been parallelized using OpenMP. However, the performance became worse with larger protein pairs because some of the MIC computing cores could not be used due to memory limitations. The overall performance was comparable to eight CPU cores, i.e., a CPU socket. These results suggest that a GPU is now more suitable than a MIC to accelerate FFT-based protein-protein docking calculations.

## List of abbreviations

GPU: graphics processing unit; CUDA: compute unified device architecture; MIC: many integrated core; FFT: fast Fourier transform; IFFT: inverse fast Fourier transform; PDB: protein data bank; MKL: math kernel library.

## Competing interests

The authors declare that they have no competing interests.

## Authors' contributions

TS, SS and MO performed the MIC implementation of protein-protein docking. TS performed the computational experiments and validated the results. TI assisted with the implementation. YA supervised and directed the entire study. TS and MO wrote the manuscript. All authors read and approved the final manuscript.

## Declarations

The publication fee of this article was funded by Tokyo Institute of Technology. This work was supported in part by NVIDIA CUDA Center of Excellence (CCOE), HPCI System Research Project (hp140173), a Grant-in-Aid for JSPS Fellows (23·8750, 24·8766, 26·30002), a Grant-in-Aid for Research and Development of The Next-Generation Integrated Simulation of Living Matter (ISLiM) and by the Education Academy of Computational Life Sciences (ACLS) at Tokyo Institute of Technology, all of which were from the Ministry of Education, Culture, Sports, Science, and Technology of Japan (MEXT).

This article has been published as part of *BMC Systems Biology *Volume 9 Supplement 1, 2015: Selected articles from the Thirteenth Asia Pacific Bioinformatics Conference (APBC 2015): Systems Biology. The full contents of the supplement are available online at http://www.biomedcentral.com/bmcsystbiol/supplements/9/S1
